# It’s a woman’s thing: gender roles sustaining the practice of female genital mutilation among the Kassena-Nankana of northern Ghana

**DOI:** 10.1186/s12978-021-01085-z

**Published:** 2021-03-01

**Authors:** Patricia Akweongo, Elizabeth F. Jackson, Shirley Appiah-Yeboah, Evelyn Sakeah, James F. Phillips

**Affiliations:** 1 School of Public Health, University of Ghana, Legon, Ghana; 2 Heilbrunn Department of Population and Family Health, Mailman School of Public Health, Columbia University, New York, NY USA; 3 Benmore, P. O. Box 650698, Joahnnesburg, 2010 South Africa; 4 Navrongo Health Research Centre, Ghana Health Service, Navrongo, Upper East Region Ghana; 5 School of Public Health, Columbia University, New York, NY USA

**Keywords:** Female genital mutilation, Female genital cutting, Social determinants, Gender stratification, Ghana, Sahelian Africa, Harmful traditional practices

## Abstract

**Introduction:**

The practice of female genital mutilation (FGM/C) in traditional African societies is grounded in traditions of patriarchy that subjugate women. It is widely assumed that approaches to eradicating the practice must therefore focus on women’s empowerment and changing gender roles.

**Methods:**

This paper presents findings from a qualitative study of the FGM/C beliefs and opinions of men and women in Kassena-Nankana District of northern Ghana. Data are analyzed from 22 focus group panels of young women, young men, reproductive age women, and male social leaders.

**Results:**

The social systemic influences on FGM/C decision-making are complex. Men represent exogenous sources of social influence on FGM/C decisions through their gender roles in the patriarchal system. As such, their FGM/C decision influence is more prominent for uncircumcised brides at the time of marriage than for FGM/C decisions concerning unmarried adolescents. Women in extended family compounds are relatively prominent as immediate sources of influence on FGM/C decision-making for both brides and adolescents. Circumcised women are the main source of social support for the practice, which they exercise through peer pressure in concert with co-wives. Junior wives entering a polygynous marriage or a large extended family are particularly vulnerable to this pressure. Men are less influential and more open to suggestions of eliminating the practice of FGM/C than women.

**Conclusion:**

Findings attest to the need for social research on ways to involve men in the promotion of FGM/C abandonment, building on their apparent openness to social change. Investigation is also needed on ways to marshal women’s social networks for offsetting their extended family familial roles in sustaining FGM/C practices.

## Introduction

Female circumcision is a deeply rooted custom in many African societal settings. The practice of female genital mutilation (FGM/C) occurs in 28 African countries, although national borders are less relevant to delineating zones for this practice than transnational cultural zones. According to the United Nations Children Fund (UNICEF), at least two hundred million girls and women are “circumcised” in 31 countries across three continents, with more than half of these “circumcisions” occurring in African countries [1]. Although the practice has existed for centuries among different groups, beginning in the 1990s this practice became a matter for international discussion [2–4] that continues to the present [5–9].

The determinants of FGM/C are complex, ranging from socio-cultural norms and economic factors, to health services and hygiene, religion, and gender stratification customs amongst others [10]. Gender determinants are typically emphasized by feminist commentators who argue that FGM/C is rooted in the need for men to control women’s sexuality, prevent promiscuity, ensure premarital virginity, marital fidelity and male sexual satisfaction [10]. While this discourse is neither monolithic nor unidimensional, FGM/C is widely recognized as a consequence of patriarchal oppression and the subjugation of women. In concert with this perspective, an advocate of FGM/C abandonment stated in a formative article in the 1990s that, “FGM/C is…as a culturally approved form of violence against women….” [11]. Moreover, its practice is known to have detrimental perinatal survival effects [12, 13]. This finding has fostered a series of recommendations on what must be done to end the practice and views about gender roles in sustaining FGM/C. Although three decades have elapsed since the Hosken report was disseminated, passages continue to have widespread currency nearly three decades later:

“To claim- as many African men do (as well as male, Western anthropologists)- that women are the ones who perpetuate the operations in societies where women have never had any choice about anything, least of all their bodies, is ludicrous. Clearly, it is a matter of sexual politics. As soon as men stop demanding FGM/C as a price for marriage and stop paying for having their daughters mutilated, the operations would stop” [11].

It has become the conventional wisdom to attribute gender stratification as the fundamental exogenous determinant of the practice, with many influential observers concluding that addressing the FGM/C problem requires prior social change leading to gender development. In recent years, the Western media has garnered a plethora of accounts from African women who were subject to the cruel and inhuman bodily mutilation that FGM/C represents to foster the view that African traditions of male dominance and the patriarchal system must change for the practice of female circumcision to be eliminated. As one respected commentator has noted:

“In the eyes of many educated people, female genital mutilation is the consequence of a patriarchal and polygamous society which has always sought to tame and subdue women” [14].

There is little doubt that feminist discourse on the root causes of FGM/C has correctly identified the underlying gender determinants of this harmful practice. Evidence based programmatic implications of this perspective are less clear, however. Instead, international discourse has assumed the character of exhortations for African governments to take punitive action. In several countries, particularly in Ghana, this perspective has generated reliance on laws that attempt to criminalize the excisor performance of the practice [15]. While laws may have had some impact, and comprise a needed component of public policy, it is likely that legal sanctions drive the practice underground in many settings [16–18], thereby complicating efforts to understand the practice and to address the issue openly in a manner that would lead to sustained social change. As yet, social science has contributed little to guiding policy and action. Although clarifying social dynamics associated with the practice could improve program efforts to eradicate FGM/C, deliberations on what to do about the practice have been relegated to essays, critiques or commentaries rather than to empirical research. In particular, there is a dearth of information on how sustain the practice gender roles that is derived from interaction with women and men themselves. As one writer noted:

“Research on female ‘circumcision’ not only has to take into account the place of the practice in the culture in question but also has to be foregrounded in a multifaceted analysis of the lives of those women whose genitals have become the subject of study” [19].

This paper provides qualitative data from field based study to explicate the gender dynamics of female circumcision in the Kassena-Nankana district of northern Ghana, a locality of northern Ghana that is known to have a high FGM/C prevalence [20–23]. Focus group discussions with women and men are employed to illustrate the gender roles that influence the practice of female circumcision in a rural, traditional African setting. It investigates the social, cultural, and physical mechanisms that sustain female circumcision, with particular attention to clarifying gender roles in FGM/C decision-making.

## Background

FGM/C or female circumcision is a generic term for traditional practices involving the cutting of female genitalia leading to the partial or the total removal of the female genitalia or injury to the female genital organ for cultural or any other non-therapeutic reasons [6, 24]. Four major types of FGM/C have been identified [24]. Type 1, also known as Sunna or “circumcision”, is defined as the partial or total removal of the clitoral glands and/or the prepuce/clitoral hood. This practice typically represents only a small proportion of women who undergo FGM/C and only a few ethnic groups practice it. Type 2 involves the partial or total removal of the clitoral glans and the labia minora, with or without removal of the labia majora. Type 2 is also known as clitoridectomy or excision. WHO estimates that about 85 percent of women who undergo FGM/C have experienced excision [7]. Type 3 also known as infibulation is the narrowing of the vaginal opening through the creation of a covering seal. The seal is formed by cutting and repositioning the labia minora, or labia majora, sometimes through stitching, with or without removal of the clitoral prepuce and glands. Type 4 involves all other harmful procedures to the female genitalia for non-medical purposes, e.g. pricking, piercing, incising, scraping and cauterizing the genital area. Though all the four types of FGM/C are associated with increased health risks, imminent complications can include psychological hazards including pain, trauma, and severe physical complications, such as bleeding, genital tissue swelling, urinary problems infections, or even death, as well as indirect psychological effects on women’s self-image and sexual lives [3, 25].

FGM/C is prevalent in the three northern regions of Ghana where the Kusasi, Frafra, Kassena, Nankam, Busanga, Walla, Dagaabas, Builsa and Sisala ethnic groups reside [26]. In the Upper East Region, FGM/C is prevalent in the Bawku Municipality, Pusiga District and the two Kassena-Nankana districts [23]. Although FGM/C is not a cultural practice in the southern parts of the country, migrants from the three northern regions and neighboring Sahelian countries sustain the practice it in the areas in where they have settled. The national prevalence in Ghana at the time of this study was estimated to range between 20 and 30 percent, while the combined prevalence for Upper West and Upper East regions was estimated to be 86% [4]. The prevalence of FGM/C in Ghana was recently estimated by the Ghana Multi-cluster Survey to be 4% of women of reproductive age [5, 27]. However, marked regional disparities in prevalence of FGM/C were documented in this study. In the Upper East the prevalence of FGM/C was reported at 27.8%; 41% in the Upper West and 2.8% in the Northern Region. Although the practice has declined in recent years, estimates show that FGM/C persists as a common practice [28, 29]. Although the practice is declining nationally, there are pockets of rural areas where the practice is still prevalent. In rural areas women are three times as likely to have experienced FGM/C than women living in urban areas [30]. In the Pusiga district of the Upper East region of Ghana, 62% women report having undergone FGM/C [31].

In 1995, the Navrongo Health Research Centre (NHRC) initiated research aimed at describing and understanding the practice of female circumcision in Kassena-Nankana district of the Upper East Region. Studies of various sub-groups in the district revealed that FGM/C is practiced among the Nankam, Kassem, and Builsa ethnic groups that comprise nearly all of the population of the district. A 1995 survey of households in the district revealed that 77% of 5275 randomly selected women of reproductive age had undergone FGM/C. In 1995–1996, a clinic-based study of 398 pregnant women seeking prenatal care found that all three types of FGM/C (circumcision, excision, and infibulation) are practiced in the Kassena-Nankana district. The majority of the women (62%) were circumcised between 15 and 19 years of age, and by age 20, 80% had already undergone FGM/C [13]. In a more recent study, 29% of 5071 deliveries in the Kassena-Nankana district were FGM/C associated. Additionally, the prevalence of FGM/C was 61.5% was among women who were over 40 years and 14.4% among those less than 20 years [32].

Among the Kassena-Nankana, FGM/C usually takes place after puberty and before marriage to mark the beginning of womanhood. After the harvest of the early millet in August, circumcision is organized at the clan or village level. Although, age is a consideration in determining eligibility for FGM/C, other factors such as impending marriage, development of breasts, early menstruation and growth of pubic hair influence the age at circumcision.

Religious belief systems are also a factor in FGM/C decision-making. Traditional religion among the Kassena-Nankana involves a process whereby an extended family patriarch consults with a shaman, termed a “soothsayer” for the purpose of contacting ancestral spirits to explain the past, interpret the present or forecast the future on matters of current concern to his lineage [33]. Often a girl’s father or extended family head consults with a “soothsayer” to determine if ancestral sprits designate a girl as being ready for circumcision or eligible for exemption from the practice. Thus, the rites of traditional religion may exempt a girl from circumcision [33]. However, more typically, beliefs about FGM/C are embedded in traditional religion in ways that support the practice. The *piligo* (in Nankam) and *sogo* (in Kassim) is a terra cotta pottery bowl that is broken in the middle of the funeral procession of a deceased woman by the daughter. It is first, a sign of farewell to the woman. Secondly, it shows that the woman was fertile in her lifetime and had children. Most importantly, the breaking of the ‘piligo’ (or sogo) is believed it is to enable the deceased woman to continue her duties as a woman in the next world. A woman who does not have a daughter to do this for her is believed to roam in the ancestral world hungry and thirsty and is not welcomed by her ancestors.

Discussion of FGM/C in Kassena-Nankana district reveals that adult women and men justify the practice as a mechanism that is believed to instill societal morals and values in a young girl before she assumes a larger role in the community as wife and mother. Traditionally, after the harvest of the early millet in August, girls who are deemed physically mature are organized to undergo circumcision by an excisor who visits the various villages. FGM/C is mainly performed on girls ranging in age between four and 14 [34]. Studies suggest that most Ghanaian girls who undergo the procedure are were under age five when FGM/C is conducted [26]. However, in some cases, FGM/C is performed on girls immediately prior to marriage [34]. Elderly women who are present at the circumcision ceremony will encourage the young girls to be brave during the procedure. Afterwards, the girls are assembled in one compound under the guidance of two or three elderly women who nurse their sores while providing education on societal values, norms and morals. The girls who have undergone the circumcision prior to marriage are taught how to cook and how to be a wife and member of their husband’s extended family [13]. At menarche life skills are discussed and the community members are expected to recognize circumcised young woman as a person who is allowed to receive marriage proposals.

## Methods

The Navrongo Health Research Centre launched an experimental study to test hypotheses about means of reducing the incidence of FGM/C through social action and community outreach. The Navrongo program developed in collaboration with local government and non-governmental organizations to change the behaviors and attitudes associated with FGM/C through education to all sectors of the community and the provision of livelihood activities for adolescent girls. A cohort of adolescent girls in six villages were exposed to different social action programs and observed over a five years period of time [35]. The current study was the component of this investigation that provided a baseline appraisal of social determinants of FGM/C in study areas.

*Data collection*. This paper is based on the analysis of twenty-two focus group discussions with men and women in 11 communities of the Kassena-Nankana district of northern Ghana: Mirigu, Paga-Bagtua, Chiana-Katiu, Natugnia, Pungu, Nayagnia, Gomongo, Mayoro, Janania, Gongnia, Chiana-Kayoro. Sessions were convened prior to the implementation of social interventions for mobilizing community support for FGM/C eradication. Since open discussions of FGM/C with different groups in the community is common, focus group discussions (FGDs) were deemed to be an appropriate form of eliciting views on the topic from a wide array of social groups.

Categories of potential respondents could be readily compiled for this study for Demographic Surveillance System (NDSS) at the Navrongo Health Research Centre (NHRC) system delineates groups of extended families compounds into clusters that were stratified into zones from the north, south, east and west sub-districts of the district for random selection, based on NDSS household enumeration numbers [36–38]. Based on randomly selected household numbers, Field staff of the NDSS, known as a Community Key Informants (CKIs) identified the precise location of the selected compounds was known. NDSS staff visited sample households and invited members to participate in focus groups according to designated gender and age groups represented by each session. This procedure was intended to identify at least one participant per compound. Sample substitution was unnecessary because all sample compounds yielded one or more individuals who agreed to participate in discussion sessions. Two focus group discussions comprising a male and female group each was held in each community. Written and oral informed consent was sought from participants who participated in the study. A total of 22 focus groups were selected, each ranging in size from 8 to 10 participants. The interviews were conducted either in Kassim or Nankam, the two main language groups in the district. The interview guides were developed in English and the translated into the two languages and back translated into English to ensure translation accuracy.

Participants for fifteen focus group discussions were selected based on the following age and sex criteria defining panels of adolescent boys and adolescent girls (aged 20–24); reproductive aged men and women (aged 26–34 and 35–49) and older men and women (aged 50 and above). This classification was sufficient for representing the experiences and beliefs associated with FGM/C as they differ among age and gender groups. Participants for seven of the focus group discussions were females selected according to age and marital status categories defining polygamous unions (aged 25–35 and 35–49) and single unions (aged 25–35 and 35–49). There were two adolescent female groups and 2 adolescent male groups (20–24). Female marital status was chosen as a selection criteria based on the assumption that having co-wives will have an observed effect on circumcision status after marriage. There were 11 female groups and 11 male groups of various ages and marital status.

A moderator and a note taker were present at each discussion. The moderators’ questions and participants answers were recorded on an audiotape. The interview guides were pretested in the central zone of the district. The FGDs were conducted to obtain insights on how engrained the practice of FGM/C was, social norms governing and sustaining the practice, perceptions of change in the practice and the extent of participant’s understanding of the health risks and law banning the practice of FGM/C. Participants were also invited to discuss factors which could facilitate social action for eradicating the practice. A transcriber, who was not present at the time of the discussion, described the data from the audiotapes and translated discussions into English before analysis of the transcripts. Data were entered with a conventional word processor, stored electronically, and dispensed to the research team without labels that could identify the participants. Analysis embraced the conventions of grounded theory analysis whereby verbatim transcripts, which were coded by key words, categorized into specific themes and utilized for the extraction of key themes [39]. Quotes representing these key themes are presented in this paper. All quotes chosen were extracted in a manner that portrays discussion that was common to all of the focus group sessions. In the presentation that follows marital status has been noted with each quotation. Although quotes that are selected are chosen to portray the general view of the focus group participants, citations do necessarily represent views of all individuals. Care has been taken to ensure anonymity, although coding recorded age groups and community where sessions were convened. Occasionally words or phrases in parenthesis are added to clarify the meaning of words and statements of the quotes.

## Results

### The FGM/C decision-making process

Both men and women support the notion that there are distinctly different parental roles in the sequence of decision leading to the practice of female circumcision. Typically, the mother takes primary initial responsibility for encouraging her daughter to be circumcised:

“Your mother would always tell you that your colleagues are going to get circumcised, so you should join them. After you have gone, she would then tell your father, and he would get ready with a fowl and millet to pay the practitioner.”

—Mirigu woman, aged 35–49, single union

Thus, the father or compound heads play a role in the initial decision to undergo FGM/C and are sometimes asked to permit the circumcision, but the father’s actions are undertaken in response to decisions taken by women. Once a man is asked to sanction FGM/C, he consults with the soothsayer, and when the consultation is completed, the father or compound head informs the girl’s mother of the outcome of the consultation. Thus, while men do not initiate FGM/C decisions, their concurrence is essential. As one woman noted:

“…if the man did not support, her daughter could not be circumcised. If your father did not give his consent, you could not be circumcised…”

—Paga Bagtua, woman, aged 25–35, single union

Circumcision is performed in the post harvest season. In the months preceding the harvest, women consult with their peers in neighboring compounds to discuss plans for undergoing circumcision for girls in the community who are considered ready for the procedure. After a decision is reached to proceed with circumcision, girls are informed that circumcision rites will be performed. Respondents in all FGD panels of this study denied that any form of compulsion was employed. Instead, they stressed the view that girls are asked to decide on whether or not to undergo the procedure. It is nonetheless clear from this study that personal agency in a girl’s FGM/C decision-making is more an illusion than reality. Instead, powerful social pressure is exercised by key players: mothers, mother-in-laws, compound heads and their wives, fathers, husbands, co-wives, peers and birth attendants.

Once a group of girls have been identified the parents and community leaders extend an invitation for a local excisor to circumcise their daughters. Other families from neighboring towns and villages who hear about the arrival of the excisor may also ask that their daughters join the group of girls undergoing the circumcision. Both the mother and the father give the excisor different forms of payment. Payment may range from cash to goods. The mother is usually responsible for providing cloth, calabash, shea nuts, while the father provides cash, fowls or millet.

“….she provided fibre threads [cloth], calabashes and shea-nuts. The man only gives a fowl….”

—Paga Bagtu woman, aged 25–35, single union

“When I have my daughter, I make her get circumcised and all the fines are mine to pay. I will have to give some fowls, guinea fowls, and millet.”

—Chiana-Katiu man, aged 35–49

### The role of men

Husbands and fathers each have specific roles to play in female circumcision. Focus group discussions indicated that fathers rarely exert pressure on their daughters to be circumcised. The role of husbands is even less pronounced than the father’s role. Nonetheless, men play a significant role in the decision-making system.

*Fatherly duties* It is evident from the FGD data that men are not instigators in the circumcision of their daughters. Fathers grant permission for their daughters to undergo circumcision, but seldom encourage their daughters to undergo circumcision. When a girl is very young, her father consults with a soothsayer to determine whether or not his daughter should be circumcised. In the animist traditions of the Kassena and Nankana, women are believed to be the property of a lineage and lineal gods are the guiding spirit of all individuals. Women are not allowed to consult with soothsayers. However, men often seek spiritual guidance in religious séances. A soothsayer is a spiritual leader who performs traditional rites that are believed to establish communication with ancestral spirits. In this course of consultation, a girl may be exempted from the practice if the soothsayer consultation reveals that she belongs to a god who does not want her to be circumcised. When eligible girls are old enough for circumcision, fathers pay fees to the circumciser:

“Men do not insist at all but it is the mothers.”

—Natugnia woman, aged 35–49, polygamous union

“If there is pressure on the girls to get circumcised then I feel it is the mothers because men do not really care about the practice”.

—Pungu man, aged 26–24

Some women reported that fathers had to be consulted before the act, though in some cases, circumcision took place without the father knowing about it. In such cases, fathers are asked to pay fees afterwards. A compound head or family head may also be responsible for the circumcision fees for girls in his compound.

“It is the compound head who gives the millet and fowls to be given to the circumcisor…”

—Nayagnia man, aged 35–49

“Sometimes the compound heads do not even know that the girls have gone for the circumcision. It is when they are asked to pay some fees that they get to know about it.”

—Chiana-Katiu men, aged 35–49

*Marriage of daughters:*It is the responsibility of fathers to arrange the marriage of daughters. In the tradition of the Kassena-Nankana, a man seeking a husband for his daughter will approach fathers of young men to discuss marriage and bridewealth. In the past, circumcision status was an important prerequisite to marriage. However, this has changed:

“[Before]…it is only when the girl is circumcised that she can get married but now the men are impatient and do not wait for all the customs [FGM/C] to be done”.

—Pungu man, aged 26–34

Once a girl has been circumcised, either before or after marriage, her father is eligible to receive a full bride wealth payment.

“In the past when an uncircumcised woman got married, her parents could not claim her dowry. So if she wanted to get married then she had to get circumcised.”

—Chiana Katiu woman, aged 26–34

A father of an uncircumcised girl may have no right to bride wealth payments. Still, if a dowry is given to an uncircumcised girl, her brothers may benefit from the bride wealth while her parents do not. Since the bridewealth payments are obligatory if a bride is circumcised, economic incentives derived from bridewealth undoubtedly influence the beliefs and motives of the father and other men in the family.

Nonetheless, the economic role of FGM/C in bridewealth appears to be eroding, as suggested by the focus group discussions. It is apparent that circumcision status is less of a determining factor in the payment of bride wealth fees now that it has been in the past.

*The risks posed to the man’s extended family by wife’s sexual desires* Men voiced strong opinions about the relationship between FGM/C and female sexuality. Men often noted that circumcision was a social necessity in the past because wife’s sexual desires were a threat to harmony in the extended family.

“What I know is, our grandparents could travel for long without returning, by the time they are back, their wives would have had contact with other men. So it was done to reduce the sexual anxiety in women”.

– Nayagnia man, aged 35–49

Such beliefs and FGM/C values may be grounded in African customs of polygamous marriage and family building that is associated with weak emotional bonds between spouses and an element of spousal mistrust [40–42]. FGM/C is perceived by men as a mechanism that is needed to control sexual desires or urges (*nyane*) in women, thereby inhibiting sexual rivalry among co-wives. This is because once a woman’s interest in sexual pleasure and ability to enjoy sex is decreased, she is likely to be unfazed by the sexuality of her co-wives. In keeping with this perspective, some men stated that they wanted their wives to be circumcised to prevent sexual liaisons between their wives and other men:

“I will like my wife to go through circumcision. There is the belief that when she is not circumcised, she has ‘nyane’ (sexual urge), sometimes in her that makes her want to sleep with other men.”

—Gomongo adolescent male, aged 20–24

Men thus see women’s sexual urges as dangerous and destructive to the extended family. So extreme are sentiments about this danger that some men believe that unbridled sexual passion can even kill plants. For example, an uncircumcised woman’s *nyane* can cause calabash plants to wither and die:

“I have seen it with my naked eyes before (uncircumcised woman crossing a calabash plant and harming it). This is because since she is not circumcised, she is still a child and she has a lot of nyane in her, which destroys things.”

—Gomongo adolescent male, aged 20–24

Several discussions nonetheless revealed that this traditional view of the dangers of sexuality has shifted. Although some FGD participants believe that female sexuality is dangerous more participants believed that intercourse with uncircumcised women would be more enjoyable and exciting than intercourse with a circumcised woman.

“Some men want the two for variety, but the old type needed the circumcised ones because they were the traditionally obedient ones. Today, no man would go for a circumcised woman, because they feel they are not as exciting as the uncircumcised ones.”

—Nayagnia man, aged 35–49

Women and adolescent girls also stated men now preferred uncircumcised women:

“Most men prefer uncircumcised women especially when it comes to sexual intercourse. They claim uncircumcised women are better in bed as compared to circumcised women.”

—Mayoro adolescent woman, aged 20–24

*The husband’s contribution* Some men express the view that circumcised brides are more wholesome and more likely to be a virgin, particularly if the girl had no objection to undergoing FGM/C:

“If she refuses[to undergo circumcision], the father will say that she is not a virgin that is why she has refused to be circumcised.”.

—Gomongo adolescent boy, aged 20–24

Most men stated that they did not care if a wife was uncircumcised or not. In many cases, men do not know if their bride has been circumcised.

“When a man is going to marry a woman, who will know whether the woman is circumcised or not?”

—Janania man, aged 50 + 

Women, concur with the notion that men do not really care about FGM/C:

“…the men do not care because they are not insulted [if their wife is uncircumcised]. What he needs is the vagina, and the clitoris does not block his interest, so he does not mind whether his wife is circumcised or not.”

—Gongnia middle-aged man (age unknown)

While pressure may arise from the husband’s household, the husband himself may not have a direct influence on his wife’s circumcision status, even after marriage.

“It is because of the insults. It isn’t your husband who will insult you. It is your co-wives and your husband’s mother [who will do so]. If you are staying with only your husband, there won’t be any problem.”

—Gyanania woman, aged 35–49, polygamous union

Discussions thus suggest that for men, the various factors that explained their support for FGM/C in the past have changed in present times. While men appear to embrace the notion that a circumcised woman is valued for her fidelity this cultural perspective is increasingly offset by males’ preference for uncircumcised women.

In the Kassena-Nankana tradition, the relationship between FGM/C and girl’s chastity enhances the circumcised woman’s ability to find a husband. However, in present times, the weak significance of FGM/C for husbands has diluted the relationship between FGM/C and marriage. As many men admit their indifference towards marrying an uncircumcised or circumcised woman; and, an increasing number of younger men preferring to marry uncircumcised women, a husband should no longer expect to pay a high bride price for his bride based on her circumcision status. As discussed earlier this will in turn affect the economic incentives for the girl’s father who no longer can expect a high bride wealth for his circumcised daughter.

“…formally if you went courting and the lady wasn’t circumcised she would be told to do it now. When she is finally circumcised she would be made to choose from amongst the contestants (boyfriends), but today the men just go and take the girls away without doing the customary rites, so that is why you find men with uncircumcised wives.”

—Pungu men, aged 26–34

In summary, the role of men in sustaining FGM/C is important, but remote and eroding in the causal system. Chiefs exercise a role in deciding on the legitimacy of village activities. If an excisor visits a village for the purpose of performing circumcisions, he is obligated by custom to visit the chief. A council of chiefs and elders cannot ban the practice of FGM/C, but they can diminish access to excisors, alerting community members to the risks associated FGM/C, requiring parental travel and costs that would not otherwise arise. And they can affect the climate of opinion about FGM/C through comments at community gatherings. Male leaders are rarely proponents of FGM/C, however, men in general have a minor role in sustaining the practice. Economic incentives that are implicitly derived from bridewealth are being weakened by changes in male values and marriage preferences. As a consequence, most men in this FGD study were open to the idea of change.

### The role of women

*Maternal care* Women view their role in fostering the circumcision of their daughters as a part of their responsibility as good mothers. Therefore, mothers try as much as possible to ensure that their daughter gets circumcised so that a mother will retain a respectable status among the women in the community. A mother who arranges the circumcision of her daughter has fulfilled her responsibilities as a good mother. The practice is associated with womanhood and the readiness for marriage. Traditionally, as aforementioned, young girls learned specific skills during the circumcision ceremony. However, even in modern times, many women subscribe to the normative value of circumcision in the belief that a girl will be considered a woman only after circumcision. Therefore, a woman wants her daughter to be circumcised so that she is thought of as a responsible mother who has raised her daughters properly.

“To be precise it is the mothers who push their daughters to circumcise because if your daughter is not circumcised, the mother would be seen in the village as an irresponsible mother. So if a mother does all that is necessary for a daughter without circumcision, she has failed in bringing up her daughter well… Mothers who witness their daughters being circumcised, are made proud and respected for the good upbringing of their daughters.”

—Gongnia woman, middle aged

In polygamous households, tensions can arise if one woman has not circumcised her daughters while her co-wives have circumcised her daughters. Therefore, when a woman arranges the circumcision of her daughter, she also eludes ridicule from the co-wives and her children.

“Mothers can force their daughters to be circumcised just because her rivals [meaning: co-wives] are insulting her and her daughters.”

—Gomongo adolescent boys, aged 20–25

*Maintaining mother to daughter traditions.* Women point out that if the mother had undergone circumcision, daughters should also expect to undergo the practice. Since circumcision has existed for generations respondents did not see any reason why girls today should not be circumcised if their mothers and grandmothers were able to endure the practice. Elderly women consistently support the practice. This is illustrated by the following statement:

“In the past, we were circumcised, but today we have been told that we should not circumcise our daughters. To me, I think the practice is good. Our grandmothers have all been circumcised, so why should it be stopped now?”.

—Chana-Kayoro woman, aged 50 + 

“I think it [FGM/C] should be continued. I have no clitoris so why is it that my daughter should not be circumcised? My daughter will be circumcised.”

—Minigu woman, aged 50 + 

The younger women held the contrasting view that, from their experience, there are health risks associated with the procedure.

I have undergone FGM/C, but I will not advice any girl to undergo it following the rumours we hear these days. Today, it is death, tomorrow, it is loss of blood and so on.

—Mayoro adolescent female 20–24 years

Moreover, participants noted that parents who compel daughters to undergo FGM/C are liable for arrest if law enforcement officers are aware of this action:

I think that policemen should be brought here to arrest practitioners and parents who want to force their daughters into FGM/C.

—Natugnia adolescent female 20–24

If most girls are educated, they could explain to their fathers about the law banning circumcision and this would make them afraid of going to jail. So no girls will be forced to undergo FGM/C. Mayoro adolescent female 20–24.

*Maternal funeral rites.* Many women feel it is especially important to circumcise their first-born girls because a first-born girl plays an important role in her mother’s funeral rites. Among the Kassena-Nankana, daughters carry their deceased mother’s personal effects in the funeral procession. Her personal effects will include the *piligo*, which is a pot that a woman keeps as a safe for all her valuable and emergency items. Only circumcised girls are allowed to participate in her mother’s burial. For this reason, mothers often insist that her first-born daughter is circumcised for to be buried without a daughter’s participation would bring shame on the family. As one woman stated, her mother insisted on having her circumcised so that her that her own funeral rites will be proper:

“I got circumcised because my mother wanted me to take an active part in her funeral preparation when she dies.”

—Natugnia woman, aged 35–49, polygamous union

But, once the eldest daughter is circumcised a woman will not be so concerned about arranging the circumcision of her other daughters.

“The mother also sees it as very necessary for her first daughter to undergo FGM/C. She will not be much worried if only her first daughter undergoes FGM/C, leaving the younger ones.”

—Mayoro adolescent woman, aged 20–24

In contrast, male groups discuss the significance of undergoing circumcision, not in respect to parental burial rites but for rather for the girl’s own burial and ultimately, her role in the after life:

“Another tradition is that, when the woman dies uncircumcised, she would be sent off without household accessories like, calabashes and pots.”

—Paga male, aged 50 + 

“It is believed that when a woman dies uncircumcised, she would be buried like a man, to prevent that they circumcise, that is what I have also heard.”

—Nayagnia male, aged 35–49

*Marital life.* In the parental home, kin, parents, or peers can exert pronounced social pressure on a girl to undergo circumcision. Once a woman is married, however, the FGM/C opinion leaders in her father’s compound cease to have any influence whatsoever. Instead, the women of the husband’s home often have an even greater influence on the decision to undergo circumcision than her parents had during her adolescence. As the following statements suggest, the pressure to undergo circumcision can be unbearable for a young woman who has yet to have children.

“Maybe the pressure at her parent’s home to get circumcised was not very great. But in the husband’s home, the mother-in-law and co-wives would not take it easy, they will insult her thus pushing her to get circumcised.”

—Natugnia woman, aged 35–49, polygamous union

“It is always from your mother-in-law and co-wives. They will insult you in such a way that even if you think you will die when you do it, you will still do it.”

—Gyanania woman, aged 35–49, polygamous union

About one-third of marriages in the Kassena-Nankana district are polygamous and many women in monogamous unions anticipate the eventual onset of polygyny. Women are particularly sensitive to pressure from co-wives to undergo circumcision, which often takes the form of circumcised co-wives flaunting their status and openly insulting uncircumcised women in their compound. This can create a tension in the household, which an uncircumcised woman feels she can mitigate by undergoing the procedure. She may seek to undergo FGM/C even if she has married a husband who harbors no particular interest in her circumcision status:

“Look! My friends, co-wives can force you to circumcise…I have seen co-wives being mishandled because they are not circumcised. So the impact is felt more from the co-wives.”

—Natugnia woman, aged 35–49, polygamous union

Within the husband’s house, an uncircumcised woman living among the circumcised is not even considered a woman. Any slight provocation will incite many insults referring to her circumcision status. This limits her ability to assert her role in the husband’s house and challenge her rights as a married woman.

“Rivals [a term connoting dysfunctional co-wife relationships] see their uncircumcised colleagues as not being women and at the least provocation she insults her ‘Momte giee’ [translation: protruding clitoris]… Rivals would also not give you any rest but would always be insulting you. In fact, there are times that a newly married woman cannot go to the backyard garden because there is that belief that when she crosses a calabash plant it would die or it would not bear fruits just because she is not circumcised.”

—Gongnia woman, 35–49

In general, it should be noted that the pressure is particularly difficult to fend off when a junior wife is living with co-wives. A married woman’s decision to undergo circumcision is viewed by discussants as not a practice done under duress, but is a decision women take in response to peer pressure from co-wives or the wife of the compound head:

“I feel the impact to circumcise is greater from the peer group. If you are married, [pressure is from]your fellow wives. If you are a girl, your colleagues. The rest can complain [about FGM/C to you] but they cannot force you.”

—Natugnia woman, aged 35–49, polygamous union

In most households, a married woman can expect to live and spend considerable time with her mother-in-law. A mother-in-law who values circumcision is likely to ostracize an uncircumcised daughter-in-law. A mother who is circumcised usually wants her son to marry a woman who is circumcised in the belief that she will be a good, upstanding wife for her son.

“The mother-in-laws would not leave you alone, they keep attributing every bad thing in the compound to you having a clitoris. So by all means to become free of those blames, you simply [get] circumcised.”

—Natugnia woman, aged 35–49, polygamous union

“It is the husband’s mother who brings about the whole problem. When she insults you and you cannot endure it you will have to get circumcised.”

—Mirigu woman, aged 50 + 

Thus, an uncircumcised woman can face daunting social pressure from other married women. Older women, and most particularly wives of compound heads, exert a profound influence on FGM/C decision-making among all women in the compound. During childbirth, birth attendants are commonly the older women in the house. Older women who assist in delivery often attribute problems associated with delivery to not undergoing circumcision and sometimes spread rumors about the circumcision status of young mothers:

“Some circumcise after marriage because when she is in labor, the attendants say they saw a piece of wood and not a child. Some will also say they saw two children. Thus, out of anger, she could undergo circumcision.”

—Gomongo adolescent male, aged 20–24

“You can not tell from their faces, but when she becomes pregnant and is in abor, those who would attend to her would know and through that everybody in the village would get to know.”

—Gongnia woman, 35–49

*Peer support for FGM/C.* Women of all ages expressed the view that circumcision is prerequisite for peer social acceptance in extended households. This sentiment becomes manifest in household discussion of circumcision as a mark of womanhood. An uncircumcised woman is alienated among women inside or outside the home, and made to feel that it is imperative that she becomes circumcised to be socially accepted. If she is not circumcised, she can expect to be socially maligned by her peer group. Among the Kassena-Nankana, girls are encouraged to eschew individualism and honor corporate familial values and group participation. Being excluded from a peer group is particularly feared, since adolescent girls have little autonomy and social interaction in highly valued. Adult women were consistent in expressing the view that peer pressure to undergoing circumcision was intense, mainly because circumcision is necessary for preventing familial discord:

“Living with circumcised women is very unpleasant, every where they go your name will be mentioned as one of the women who have not been circumcised. Mockery will be very common and you can never feel free conversing with them because you would be seen as a social outcast. That is why every woman was advised to circumcise, in order to be able to join the women fraternity.”

—Natugnia woman, aged 35–49, polygamous union

“It is true, when you are an uncircumcised woman in a group of circumcised women, you will never feel comfortable, because they keep looking down upon you. You had to get circumcised to be part of the group.”

—Chana Katiu woman, aged 25–34

Expressions of pressure to undergo circumcision after marriage, however, are sometimes less a matter of antagonism than a form of sisterly social support. “Friendly advice” maybe extended to young women by peers, a mother in law, or co-wives who warn young women about the ostracism that an uncircumcised woman will face in the future. The following statements express this view:

“When you are sick, your husband’s mother is concerned and tries to make you feel well. In the same way when you are uncircumcised, your husband’s mother gets you circumcised so that you can move and speak freely with members of the household.”

—Chana-Kayoro woman, aged 50 + 

“Sometimes, too, when other people keep insulting you, good co-wives may advise you to get circumcised.”

—Chana Katiu woman, aged 25–34

The role of peers and colleagues appears to have the greatest a influence immediately prior to marriage; and co-wives have the greatest influence immediately following marriage. Peers and colleagues may influence a girl to the extent that a girl will undergo circumcision without her parent’s encouragement or active support. Young respondents elaborated on this peer pressure to undergo circumcision.

“Sometimes the girls themselves might demand to be circumcised. This could generate from the fact that their colleagues are circumcised and thus tease them when they are together.”

—Gomongo adolescent boys, aged 20–24

“It could also be your colleagues. They can mock at you and that will compel you to go in for FGM/C since you will not like to be a laughing stock and branded as weak…They refer to you as a man and call you ‘long clitoris’. This annoys you and gives you the urge to go and get circumcised.”

—Mayoro adolescent girls, aged 20–24

While it is apparent that FGM/C is not a practice that is undertaken by overt force, it is abundantly clear that adolescent girls face daunting social pressure from mothers, other adult women in the extended family and peers. The actors in the FGM/C decision-making system change when a young woman marries, and her autonomy on this issue typically diminishes further. A young woman “acquired” through family exchanges has little status in her new household and must demonstrate devotion not only to her husband, but also to his complex extended family. Given the FGM/C-supportive social structures that young women must operate in, it is little wonder that the practice has remained pervasive. Although circumstances under which circumcision occurs vary all over African countries, the concept of force merits some clarification in this context.

Social forces that impinge on a young woman’s FGM/C motives are complex, robust and pronounced. In general, women have a more active role in sustaining various roles in the practice of FGM/C than men. As mothers, daughters, peers, and co-wives, women are socially invested in the continuation of the FGM/C practice. A mother who influences her daughter to undergo circumcision avoids ridicule of herself and her daughter and insures that her own funeral rites will be performed correctly. Similarly, peers and co-wives also avoid this derisive behavior by encouraging a woman to undergo circumcision. Once a woman undergoes circumcision, she has the right and capability to negotiate her role in her community of women and an element of dignity in her extended family that she would otherwise lack.

## Evidence of preferences that oppose FGM/C

*Health concerns.* Despite considerable evidence of the continuing social value of FGM/C, there is some indication that support for the practice is eroding and that prevalence of circumcision may be declining. It was a common theme in discussions that FGM/C is declining as part of the erosion of wholesome family values in general and the decline in FGM/C was perceived to be contributing to social malaise. Both female and male respondents nonetheless suggested positive reasons for this trend, which ranged from health concerns to the notion that circumcised women are often sought by men to the general sense that circumcision is “outdated”. Some of this change in social perception is based on misinformation, and some is based on actual factors regarding health problems associated with FGM/C. More typically, however, health concerns reflected a blend of information and misinformation. An example includes the perception that a decline in the nutritional content of food is believed to make it harder for women to recover the blood that they lose during circumcision.

“…now that the food we eat is not very nutritious as before, we can not risk wasting blood through circumcision.”

—Natugnia women, aged 35–49, polygamous union

Men who opposed the practice were sometimes cognizant of the health implications of practicing FGM/C such as the effect of FGM/C on childbearing, but expanded this notion to include child health more generally. For example:

“I prefer the uncircumcised [women] because whenever she brings forth, you will notice that the child is beautiful and healthier than the circumcised woman’s child.”

—Gomongo adolescent male, aged 20–24

These views demonstrate the need to provide effective health education about the effects of FGM/C in ways that combat misinformation about FGM/C as well as provide a better understanding of why FGM/C should be prevented.

*Ideational change* Focus group respondents often noted ways in which FGM/C norms are changing. Although this is not the predominant view some women and many men stated that FGM/C is outmoded and that women who practice it are illiterate and ignorant. This view was expressed by a middle aged Natugnia woman:

“Whether married or not, circumcision is not practiced any longer. Only the illiterates stay indoors and still circumcise. If you assemble all girls here, the majority are not circumcised.”

—Natugnia woman, aged 35–49, polygamous union

Women expressing this view also acknowledge that the pattern of mockery that was once directed to the uncircumcised is now more typically expressed as mockery against those who are circumcised. For example, a respondent claimed that women who are circumcised are now lectured about the practice when they encounter health workers.

“These days when you are circumcised and you are in labor at the hospital, the nurses insult you so much.”

—Gongnia woman, 35–49

Some women cited that they have experienced being ridiculed for having an “empty vagina:”

“These days if a circumcised woman tries to look down on an uncircumcised one, she will be seen as ignorant or even an illiterate, because the practice is outdated now. So when you are insulted that you have a protruding clitoris, also return the insult by saying that she has an ‘empty vagina’.”

—Natugnia woman, aged 35–49, polygamous union

Still, while many circumcised women wanted their daughters and other women to undergo circumcision, some professed a sense of opposition against the practice. Reasons cited were usually health related, though some circumcised women expressed regret that sexual relations were enjoyed more by uncircumcised women.

“We those who are circumcised don’t enjoy sex as much as the uncircumcised women. We never knew it was harmful to us or we wouldn’t have done it.”

—Gyanania women, aged 35–49, polygamous union

“I have been circumcised, but when I have a daughter, I will not allow her to get circumcised, we were circumcised because we were ignorant.”

—Mirigu old women, aged 50 + 

Thus, mockery among women is the main mechanism through which social pressure is exercised. Whereas ridicule was once directed to fostering FGM/C, it is now sometimes directed to deriding the practice. While fears of women’s sexuality once provided a rationale for FGM/C practice, there is evidence that sexual perceptions of uncircumcised women may be contributing to changing social acceptance of uncircumcised women.

But foremost, FGM/C no longer seems to be an issue that is encouraged by men and their preferences or dictated by their preferences. Moreover, many women are cognizant of the fact that men have become ambivalent about circumcision. Results of this investigation thus, challenge the view that women seek FGM/C in response to the dictates of men. Instead, women subscribe to the notion that circumcision is the concern of women only. Men have a role in the FGM/C decision making system; but all FGD age and gender groups lend emphatic support to the proposition that FGM/C is a woman’s matter that is sustained and promoted by mothers and mothers-in-law as one woman noted:

“The men would never open their mouths that a woman should [be] circumcised. It is a woman’s thing. The pressure comes from them.”

—Natugnia women, aged 35–49, polygamous union

## Conclusion

Since the practice of FGM/C is grounded in customs perpetuating the subjugation of women, it is widely assumed that male preferences and FGM/C beliefs are the decisive influences sustaining the practice of FGM/C in traditional societies. This investigation lends support to this perspective in the sense that the male dominated patriarchal system constrains women’s autonomy and leads to a system of social influence that a young woman is powerless to engage. But to conclude the analysis with this observation would do little to elucidate what happens in the daily lives of women that sustain FGM/C and what must be done to accelerate the erosion of this harmful practice.

## The FGM/C decision-making system

**Fig. 1:**
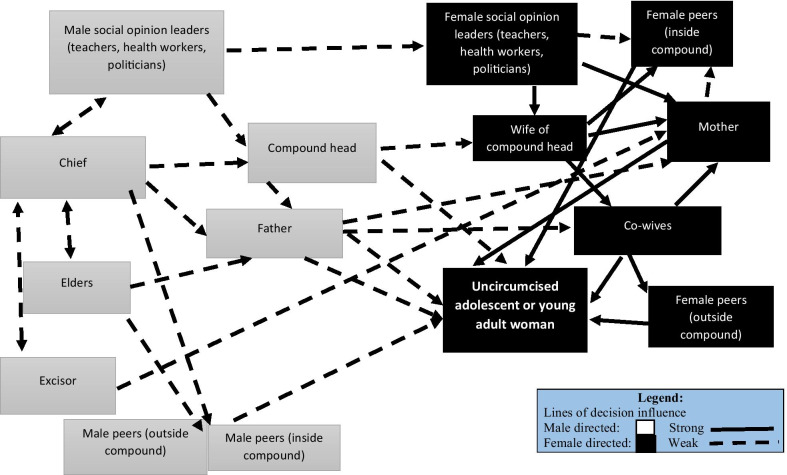
Lines of relative social influence on FGM/C decision making among adolescents

**Fig. 2:**
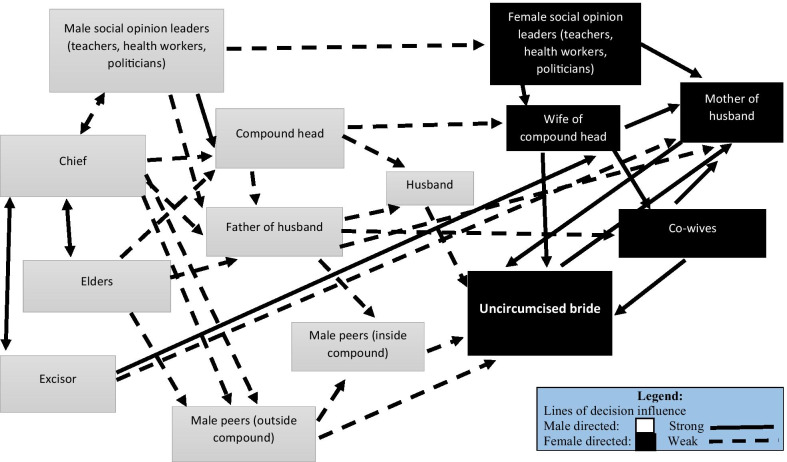
Lines of relative social influence on FGM/C decision making among newly married

The relationship of factors in the FGM/C decision-making system are illustrated in Figs. 1 and 2. Two figures are necessary in keeping with our observation that a young woman seeking to avoid FGM/C must run the gauntlet of two complex systems of social pressure, one dominated by her mother before marriage, the second dominated by her mother-in-law after marriage. As the diagrams show, most men in study communities where the practice FGM/C is extensive do not play a prominent role in FGM/C decision-making. However, the important exogeneous role of the patriarchal system should not be dismissed as inconsequential. Both men and women are players in the institution of FGM/C, but the role of women is proximate and pronounced. As the figures show, women are the main perpetrators of the FGM/C practice. Male leaders play an important role in the legitimization of FGM/C- most excisors are men and religious rites prior to the FGM/C practice are performed by men. The important, but exogenous role of men appears in the diagrams as male influences on the left hand side of the diagrams.

Figure 1 demonstrates the complex and systemic nature of social support of FGM/C. First, there is a strong component of gender stratification in the social forces that sustain female circumcision. Gender differentiation of the influences reflects the investment and benefits expected of both men and women. Fathers stand to gain monetarily from a daughter’s circumcision because of the bride wealth custom. However, the role of the bride wealth in marriage is diminishing and the value of FGM/C in determining bridewealth is eroding. These changes may be occurring at an even faster rate than change in FGM/C practice [43, 44]. Nonetheless, mothers and other women in the extended family are socially invested in their daughter’s circumcision because of the social stigma of not being circumcised. For women, the relationship between social benefits and circumcision status has not changed, while the reasons for man to marry a circumcised woman or force his daughter to circumcise has diminished. Thus, the lines of influence diagrammed in the figures are weak for men but strong for women.

The role of peers further complicates the system of influences diagrammed in Fig. 1. Girls are inculcated with the belief that group membership and corporate values are crucial to self-esteem. Ultimately, the decision to undergo circumcision thus, depends on whether or not the social climate favors being circumcised or not being circumcised. Women at all ages are immersed in a social environment that constrains social agency and perpetuates circumcision practice.

Figure 2 illustrates ways in which influences on FGM/C behavior shift with marriage. As in the Fig. 1 system, young married women have little autonomy in FGM/C decision-making, despite the common assertion that FGM/C is not compulsory. The role of extra-familial peer pressure virtually disappears with marriage and the young woman’s familial climate of FGM/C values indicated by in her husband’s extended family.

The continuing and robust influence that women play in sustaining the practice could be related to the need for women to create status for themselves in a women’s hierarchal society where power and influence is otherwise, vested in the male dominated, patriarchal system. Instead of representing an “exercise in male supremacy and the oppression of women” [45] or reasoning that women are “colluding with patriarchy to maintain subordination of women in society” [46], programs should seek to create “social space” for women that enhance the status of women that is associated with female circumcision without the actual performance of circumcision. As one writer has observed,

“to reduce adolescent girls’ belief that excision would transform them into adult women to patriarchal conspiracy would be to ignore how the institution of female initiation regulated relations among women as well as between men and women” [47].

Women could be portrayed less as victims or misguided perpetrators and more as women who can learn and create these same ideals to more positive social mechanisms and customs for themselves.

## Discussion

This study aimed at clarifying the gender dynamics that underlie social support for female circumcision in the Kassena-Nankana District of northern Ghana Results suggest that the FGM/C decision-making process is complex, involving multiple family members. However, in the course of family dynamics, the girl’s mother takes the primary initial responsibility for encouraging a daughter to be circumcised. The father’s role is also critical because he is responsible for permitting the circumcision procedure. But, the initiative for undergoing FGM/C is mainly the mother’s prerogative. This finding is consistent with conclusions reached by studies of the FGM/C decision-making process elsewhere in Africa. Qualitative research on FGM/C determinants in Sudan and Sierra Leone parallel our findings [48, 49]. Moreover, a quantitative study conducted in Iran reported that mothers and grandmothers were the main decision makers in the circumcision of 85.1% of the study participants [50]. Patriarchy is nonetheless important. A possible explanation of the role of mothers or females in perpetuating FGM/C is likely to be influenced by their desire to maintain tradition and despite the dominant role of women in sustaining FGM/C, the practice cannot be disassociated from the more general context that patriarchy conveys. Social institutions in general are grounded in patriarchal context of Kassen-Nankana society [48],

Various studies have shown that a wide range of factors have been instrumental in reducing the prevalence of FGM/C [10, 31, 32, 50]. In this study, respondents have attributed the decline in the prevalence of FGM/C to general concerns about the health risks associated with the practice, particularly pertaining to loss of blood, and the effects of FGM/C on childbearing and child health. Other factors accounting for the change in the receptiveness of FGM/C are linked to women being ridiculed for being circumcised, the general notion that sexual relations are enjoyed more by uncircumcised women and men’s increased preference for uncircumcised women. The concerns on the health risks associated with FGM/C might be due to the extensive focus on the health risk of FGM/C in numerous FGM/C abandonment interventions [48, 51]. The horrific experiences of circumcised women might have also played a role in strengthening the advocacy against FGM/C.

## Conclusion

Since the practice of FGM/C is grounded in customs perpetuating the subjugation of women, it is widely assumed that male preferences and FGM/C beliefs are the decisive influences sustaining the practice of FGM/C in traditional societies. This investigation lends support to this perspective in the sense that the male dominated patriarchal system constrains women’s autonomy and leads to a system of social influence that a young woman is powerless to confront. But to conclude the analysis with this observation would do little to elucidate what actually happens in the course of the FGM/C decision-making process. Women promote, sustain, and initiate discussion of FGM/C in the household. For programs to effectively accelerate the erosion of this harmful practice, women’s advocacy of the practice must be in focus. What then can we conclude about the appropriate design of an FGM/C prevention program?

First, since the social forces that sustain FGM/C are complex and systemic, no one strategy or simple initiative will work. Just as FGM/C is sustained by a complex social system, prevention must be guided by a sophisticated sense of respect for the institutions that govern life, prevent social disorder, and sustain family values. It is particularly important to focus on the FGM/C motives of adults; program oriented to adolescents alone will fail.

Second, instituting change is possible. Social change in FGM/C values is already evident. Program action is not a hopeless endeavor. Lines of influence that were strong in the past, such as fathers, husbands and compound heads, appear to be eroding.

Third, there is a need to build on the receptive audience that men represent. Chiefs, elders and other male players in the patriarchal system can be active promoters of FGM/C prevention. The Navrongo experiment, for example, utilizes traditional village gatherings, known as durbars as mechanisms for communicating FGM/C lessons and correcting misinformation. It is more appropriate to utilize the lineage and chieftaincy system for disseminating health education about FGM/C than professional health workers who are engaged in ambulatory care. Rather than dismissing the patriarchal system as the social force that sustains FGM/C, the system of male social leadership and communication should be marshaled to foster abandonment of the practice.

Fourth, social interaction among women is dominated by exchanges in the extended family. There is a need to build extra-familial women group identity and social cohesion around activities that challenge traditional FGM/C views. Women who are opposed to FGM/C need social support for their perspective. Singing and dancing groups exist in all of the FGM/C experimental areas. Convening such groups for the purpose of fostering FGM/C prevention, would build a program around the strong value that women consign to group participation and collective decision-making. An effective program of mobilizing women’s extra-familial networks would offset the isolation and traditionalism that constrains the autonomy of young women.

Fifth, the needs of unmarried and married adolescents for FGM/C prevention programs cannot be separated from adolescent health needs more generally. Activities that build self-esteem and autonomy through livelihood training, peer leadership, or other adolescent outreach program, such as sport promotions, can include FGM/C educational components. Girls organized into peer groups for the Navrongo FGM/C experiment had no prior experience with extra-familial decision-making or discussion of matters involving individual preferences and personal autonomy. Building peer leadership for reproductive health is a crucial element of the Navrongo FGM/C eradication strategy.

Finally, adolescent outreach activities can be designed to have a “right of passage” component whereby young men and women receive traditional family life education and their completion of this process is acknowledged by a community celebration. In this manner, elements of social values that are so often cited as rationale for sustaining the practice of FGM/C can be re-associated with a program that is designed to foster prevention of this practice. Participants in this study were generous with advice on how this could be achieved through appropriate outreach to parents, women’s groups and community leaders. While the specifics of these recommendations may not transfer to other social groups that practice FGM/C, the general principle of consulting communities, seeking their advice, and implementing a socially informed program should apply to other settings. It is possible that other investigations may challenge conventional wisdom on what must be done to prevent FGM/C. At least in this setting, men are logical allies in instituting change; and women must be the focus of social intervention if efforts to institute change are to succeed.

## Data Availability

Data for study is available upon request.

## Abbreviations

CKI: Community key informants
FGD: Focus group discussion
FGM/C: Female genital mutilation
NDSS: Navrongo demographic surveillance system
NHRC: Navrongo Health Research Centre
IRB: Institutional Review Board
UNICEF: United Nations Children’s Fund
WHO: World Health Organization
